# The landscape of circular RNA in preterm birth

**DOI:** 10.3389/fimmu.2022.879487

**Published:** 2022-08-22

**Authors:** Yuxin Ran, Ruixin Chen, Dongni Huang, Yan Qin, Zheng Liu, Jie He, Youwen Mei, Yunqian Zhou, Nanlin Yin, Hongbo Qi

**Affiliations:** ^1^ Women and Children’s Hospital of Chongqing Medical University (Chongqing Health Center for Women and Children), Chongqing, China; ^2^ Chongqing Key Laboratory of Maternal and Fetal Medicine, Chongqing Medical University, Chongqing, China; ^3^ Joint International Research Laboratory of Reproduction and Development of Chinese Ministry of Education, Chongqing Medical University, Chongqing, China; ^4^ Department of Gynecology and Obstetrics, West China Second Hospital, Sichuan University, Chengdu, China; ^5^ Department of Gynecology, The First Affiliated Hospital of Chongqing Medical University, Chongqing, China; ^6^ Department of Obstetrics, The First Affiliated Hospital of Chongqing Medical University, Chongqing, China; ^7^ Center for Reproductive Medicine, The First Affiliated Hospital of Chongqing Medical University, Chongqing, China

**Keywords:** circular RNA, preterm birth, maternal-fetal tissues, immune-inflammation, microRNA

## Abstract

**Background:**

Preterm birth (PTB) is a multifactorial syndrome that seriously threatens the health of pregnant women and babies worldwide. Recently, circular RNAs (circRNAs) have been understood as important regulators of various physiological and pathological processes. However, the expression pattern and potential roles of circRNAs in PTB are largely unclear.

**Methods:**

In this study, we extracted and analyzed the circRNA expression profiles in maternal and fetal samples of preterm and term pregnancies, including maternal plasma, maternal monocytes, myometrium, chorion, placenta, and cord blood. We identified the circRNAs which is associated with PTB in different tissues and explored their relationships from the perspective of the overall maternal-fetal system. Furthermore, co-expression analysis of circRNAs and mRNAs, target microRNAs (miRNAs), and RNA-binding proteins (RBPs), provided new clues about possible mechanisms of circRNA function in PTB. In the end, we investigated the potential special biofunctions of circRNAs in different tissues and their common features and communication in PTB.

**Results:**

Significant differences in circRNA types and expression levels between preterm and term groups have been proved, as well as between tissues. Nevertheless, there were still some PTB-related differentially expressed circRNAs (DECs) shared by these tissues. The functional enrichment analysis showed that the DECs putatively have important tissue-specific biofunctions through their target miRNA and co-expressed mRNAs, which contribute to the signature pathologic changes of each tissue within the maternal-fetal system in PTB (e.g., the contraction of the myometrium). Moreover, DECs in different tissues might have some common biological activities, which are mainly the activation of immune-inflammatory processes (e.g., interleukin1/6/8/17, chemokine, TLRs, and complement).

**Conclusions:**

In summary, our data provide a preliminary blueprint for the expression and possible roles of circRNAs in PTB, which lays the foundation for future research on the mechanisms of circRNAs in PTB.

## Introduction

Preterm birth (PTB) is defined as birth before 37 weeks of gestation. Approximately 15 million babies are born prematurely each year worldwide, which means the incidence of PTB is 10.6% (1). Since PTB is the main cause of perinatal morbidity and mortality, it has become a major public health problem that needs to be solved urgently (2). Globally, about 35% of neonatal deaths are caused by PTB, and survived preterm babies have a significantly higher risk of developing serious complications such as necrotizing enterocolitis and cerebral palsy than normal babies (3). Therefore, revealing the molecular basis of PTB and thus improving its clinical management strategies is the necessary route to safeguard maternal and fetal health. Generally, PTB is recognized as a complex multifactorial syndrome involving a widespread molecular alteration throughout the maternal-fetal system (4). Unfortunately, the overall molecular changes and underlying mechanisms of PTB have not been well elucidated, which poses challenges for the prediction and treatment of PTB. Recently, the research progress of non-coding RNA (ncRNA) molecules (e.g., circRNA) has provided new ideas for the study of the mechanisms of PTB (5).

CircRNA is a special type of ncRNA molecule with a highly stable covalent ring structure and is widely distributed in the body (6). Increasing evidence proved that circRNAs are involved in complex and diverse biological processes through multiple mechanisms, including acting as miRNA sponges, regulating host genes and downstream mRNAs, and interacting with RNA binding proteins (RBPs) (7, 8). Meanwhile, given its unique stability and tissue specificity, circRNA is regarded as a promising biomarker for predicting the occurrence and status of diseases (9). Therefore, since 2015, the investigation of the circRNA expression landscape in various human tissues (e.g., brain, heart, placenta) has attracted great attention and progressed rapidly, which has laid the necessary foundation for future circRNA studies in a variety of diseases (10–12). For example, the circRNA expression landscape in brain tissues of autism spectrum disorder (ASD), a neurodevelopmental disease based on immune-inflammatory dysregulation, has been elucidated, providing a blueprint and constructive guidelines for further research of circRNA mechanisms in this disease (10, 13). Besides, integrated analysis of circRNA expression in multiple tumor tissues provided the macroscopic circRNA landscape in cancer, which contributes to subsequent in-depth exploration of circRNAs as diagnostic or therapeutic targets across cancer types (14). In 2020, our team took the lead in exploring the circRNAs expression profile of maternal peripheral blood in PTB and demonstrated that the PTB-related circRNAs participate in its core immune-inflammatory mechanisms (5).

Based on our previous findings and the characteristic of systemic pathological changes in PTB, we believed that circRNAs are widely altered in tissues of PTB and are involved in its underlying biological processes. However, the expression landscape and potential functions of circRNAs in this complex disease remain largely unknown. To reveal the overall expression status of circRNA and its possible effects in PTB, we identified and analyzed for the first time its expression profile in maternal plasma, maternal monocytes, myometrium, chorion, placenta, and cord blood, and performed downstream predictive functional analysis from different perspectives. Our study will provide the necessary global knowledge and outline to explore the roles of circRNA, a potent and emerging molecule, in PTB.

## Methods and materials

### Data collection and evaluation

RNA-seq raw data of various tissues from term and preterm pregnancies were obtained from NCBI Gene Expression Omnibus (GEO) (https://www.ncbi.nlm.nih.gov/geo/) and NCBI sequence Read Archive (SRA) (https://www.ncbi.nlm.nih.gov/sra/) databases. Inclusion criteria for the term group were delivery after 37 weeks of gestation, whereas that for the preterm group were spontaneous labor and delivery without medical termination of pregnancy before 37 weeks of gestation. Exclusion criteria for all participants included multiple pregnancies, clinical chorioamnionitis, macrosomia, preeclampsia (PE), fetal growth restriction (FGR), and gestational diabetes mellitus (GDM). Based on these criteria, a total of 7 RNA-Seq datasets from maternal plasma, maternal monocytes, myometrium, chorion, placenta, and cord blood were ultimately included in this study, containing 63 PTB cases and 67 controls. The characteristics of all datasets were shown in **Table 1**.

**Table 1:** Summary of the characteristics of all included datasets.

Sample Type	Database	ID	Contributors	Submission date	Preterm	Term	
Maternal plasma	SRA	SRP130149	Stanford University	2018	5	5
Maternal monocytes	GEO	GSE96077	Lye S, Price N	2017	10	29
Myometrium	SRA	ERP116770	University of Liverpool	2020	6	6
Chorion	SRA	SRP139931	Facultad de Medicina Universidad de la Republica	2018	4	4
Placenta	GEO	GSE174415	Lien Y	2021	16	16
Cord blood	GEO	GSE176127	Gomez-Lopez N	2021	22	–
	GEO	GSE185557	Gomez-Lopez N	2021	–	8

### Identification and analysis of circRNA expression

The tools, CIRIquant (https://github.com/bioinfo-biols/CIRIquant) and CIRCexplorer2 (https://github.com/YangLab/CIRCexplorer2), were used to identify circRNA. Firstly, based on the back-spliced junction (BSJ), circRNAs were identified and quantified from RNA-seq raw data using CIRIquant, which is an efficient multithreading detection tool (15). This process was completed by the code recommended in its instruction document and all settings were left default. Secondly, an independent circRNA identification work was performed using CIRCexplorer2 with default settings (16). This process consists of three steps: alignment, parsing, and annotation (**Supplementary Figure 1**). Finally, circRNAs presented in both CIRCexplorer2 and CIRIquant results and detected in at least half of the samples (or in more than five samples) were selected as identified circRNAs and used for the downstream analysis. Their expression levels depended on the results of CIRIquant. In both independent identification processes, *.fastq files were used as input files, and alignment was done against the UCSC human reference genome (hg19) and gencode.v22.annotation.gtf.

The differential expression analysis was performed using the R package “limma” and the significantly differentially expressed circRNAs (DECs) were selected using |log2 (fold change)| > 1 and adjusted p-value < 0.05 as criteria. The “removeBatchEffect” function was used to correct the batch effects between datasets (17).

### Prediction of target miRNAs of DECs

Based on the potential miRNA binding sites, we predict target miRNAs of DECs *via* the TargetScan (http://www.targetscan.org/) and the miRanda (https://anaconda.org/bioconda/miranda) databases, with a threshold of total context++ score ≤ -0.60 (18, 19).

### Correlation analysis of DECs and mRNAs

Through Pearson’s correlation analysis using the corr.test function in R package “psych”, the correlations between expression levels of DECs and mRNAs were measured. Then, the co-expressed mRNAs of DECs were identified using |r| > 0.8 and adjusted p-value < 0.05 as screening criteria.

### Functional enrichment analysis

The potential functions of circRNAs were predicted through the Gene Ontology (GO) and the Kyoto Encyclopedia of Genes and Genomes (KEGG) pathway analyses based on their target miRNAs and co-expressed mRNAs in different tissues. Specifically, the functional annotation of target miRNAs was performed using the miEAA tool (https://ccb-compute2.cs.uni-saarland.de/mieaa2) (20), and that of co-expressed mRNAs was conducted by the R package “clusterProfiler” (version 3.16.1) (21).

### Construction of the circRNA-RBP networks

RBPs of DECs were predicted independently through the circAltas (http://circatlas.biols.ac.cn/) and CSCD2 (http://gb.whu.edu.cn/CSCD2) databases, respectively. The intersection of their results was considered as the candidate RBPs (22, 23). Then, circRNA-RBP networks were constructed to show their interaction using Cytoscape (version 3.6.1) software (24, 25). The connectivity degree of each node was calculated and ranked from largest to smallest for selecting the top 10 RBPs.

### Gene set variation analysis (GSVA)

This work was completed using the R package “GSVA” with the mRNA expression matrix as the input file. The *.gmt files of immune-inflammatory processes were obtained from the Molecular Signatures Database (http://www.gsea-msigdb.org/gsea/msigdb). The specific geneset enrichment scores of immune-inflammatory processes in each sample were calculated, which reflect the variation of pathway activity over a sample population.

### Statistical analysis and data visualization

The statistical analyses (t-test and linear regression analysis) were performed using SPSS software (version 25.0, IBM, Chicago, IL, USA). The partial least squares discriminant analysis (PLS-DA) was completed by utilizing the R package “ropls” (version 1.24.0) (26). All analysis results were visualized using R (version 3.6.2) and Cytoscape (version 3.6.1) software.

## Results

### Overview of circRNAs expression in preterm and term pregnancies

**Figure 1:**
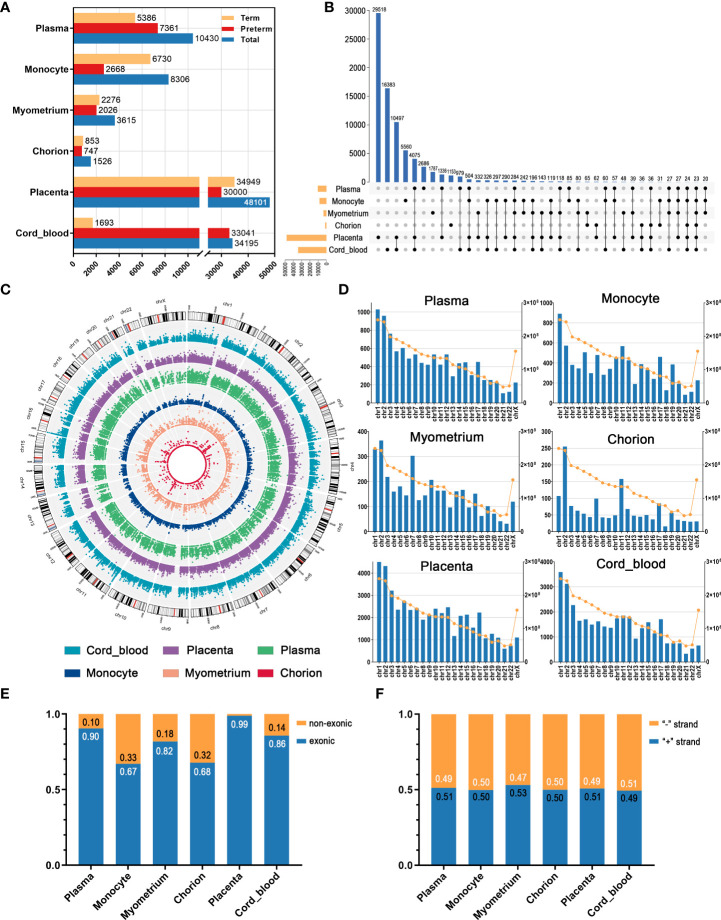
The overview of circRNAs in maternal plasma, maternal monocytes, myometrium, chorion, placenta, and cord blood of preterm and term pregnancies. **(A)** The number of circRNAs in different groups and tissue types. **(B)** The intersection of circRNAs in various tissues. **(C)** The chromosomal distribution patterns of circRNAs in different tissues. **(D)** The length and circRNA number per chromosome. The blue bars indicate the circRNA number (left Y axis) and the yellow dots indicate the chromosome length (right Y axis). **(E)** The proportion of the non-exonic and exonic circRNAs. **(F)** The ratio of sense and antisense strand circRNAs.

We revealed circRNA expression profiles of the different samples from the maternal circulation to maternal-fetal interface to fetal circulation in preterm and term pregnancies and analyzed their patterns and characteristics. Overall, the number of circRNAs varied significantly among tissues. For example, there were 10430 circRNAs in maternal plasma, while 8306 in monocytes. Furthermore, in each tissue, the number of circRNAs in the preterm group was different from that in the term group (**Figure 1A**). In addition to quantitative differences, on the whole, the individuals of detected circRNAs differed in these tissues, as manifested by the fact that most circRNAs expressed in only one tissue type. This is consistent with the well-recognized tissue specificity of circRNA (27). Nevertheless, we still noticed that several circRNAs are shared by multiple tissues. For instance, there were 284 identical circRNAs in maternal plasma, maternal monocytes, myometrium, placenta, and cord blood (**Figure 1B**). This interesting phenomenon may be related to the fact that pregnancy is a commonly acknowledged multi-systemic and multi-tissue collaborative process.

As shown in **Figure 1C**, the chromosomal distribution patterns of circRNAs in different tissues have a certain degree of similarity. In all six tissues, the number of circRNAs gradually decreased from chromosome 1 to X, consistent with the decreasing trend in the length of chromosomes (**Figure 1D**). Collectively, most circRNAs were exonic circRNAs and only a few circRNAs were non-exonic circRNAs, including circular intronic RNAs (ciRNAs) and intergenic circRNAs. In these tissues, non-exonic circRNAs accounted for the highest proportion in maternal monocytes, at 33%, whereas only 1% in the placenta (**Figure 1E**). Moreover, the proportion of circRNAs derived from the sense strand and the antisense strand was similar in all tissues, approximately 50% (**Figure 1F**).

### Key circRNAs and their potential functions in maternal plasma of PTB

**Figure 2:**
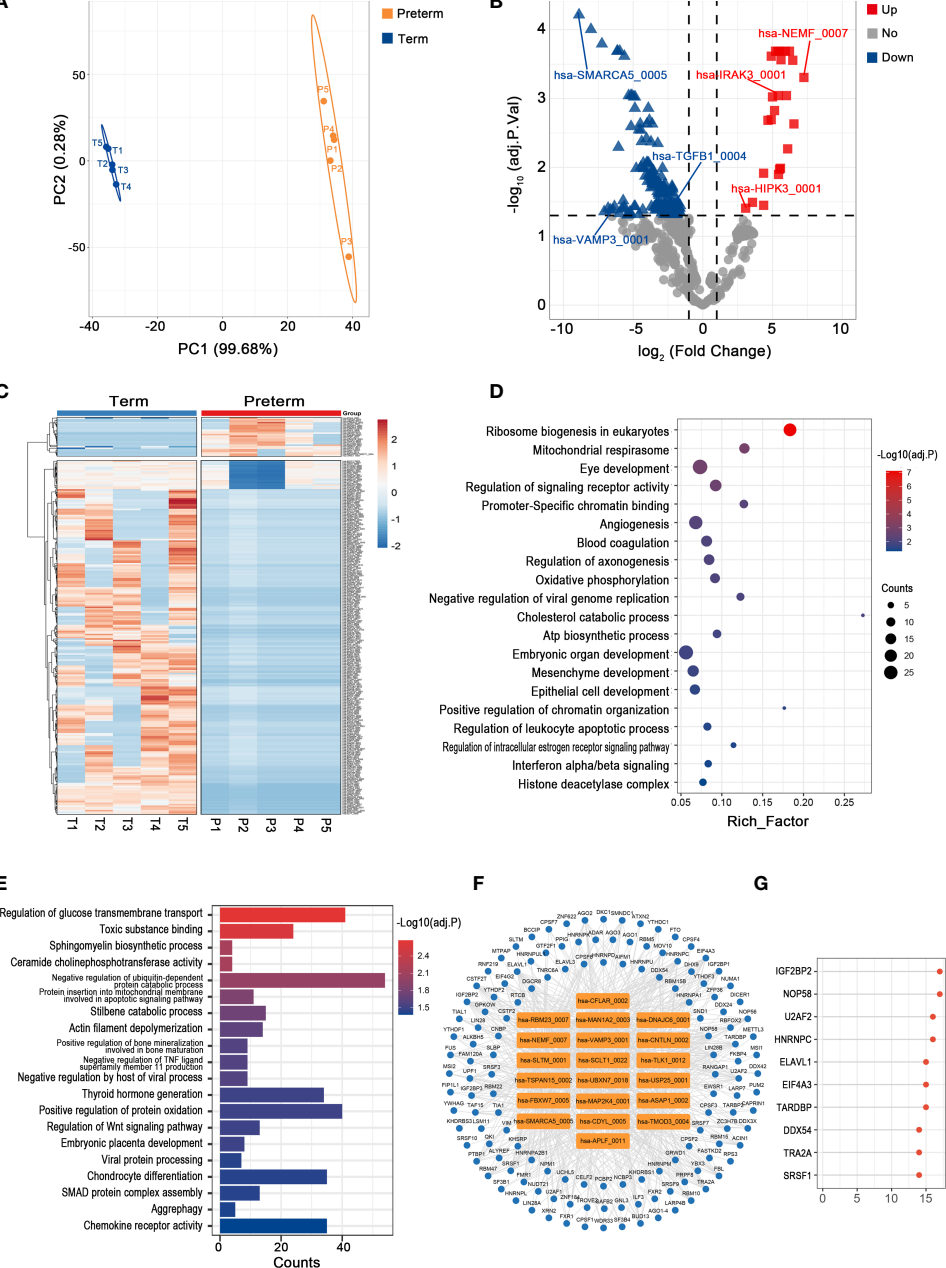
The circRNA changes and functional analyses in maternal plasma of PTB. **(A)** PLS-DA of circRNA expression in preterm (yellow points) and term (blue points) maternal plasma. **(B)** The volcano plot of circRNA changes and adj. p-values in maternal plasma of PTB. **(C)** The hierarchical clustering of DECs expression in term and preterm maternal plasma. (DECs: |log2 (fold change)|> 1 and adj. p-value < 0.05). **(D, E)** Functional enrichment analysis for co-expressed mRNAs **(D)** and target miRNAs **(E)** of DECs in preterm maternal plasma. **(F, G)** The DECs-RBPs network and the top 10 RBPs in maternal plasma.

Plasma circulates throughout the body, providing a valuable window for monitoring systemic changes in the mother. PLS-DA revealed that preterm maternal plasma samples formed a separate cluster from controls based on circRNA expression (**Figure 2A**). Here, a total of 261 circRNAs were significantly differentially expressed in PTB. Among them, 26 circRNAs such as hsa-HIPK3_0001 were up-regulated, while the remaining circRNAs were down-regulated, the top one of which was hsa-SMARCA5_0005 (log2FC = -8.88, adj. p < 0.001) (**Figures 2B, C**) (**Supplementary Table 1**).

Recent studies have demonstrated that circRNAs might regulate the expression of their host genes, but curiously, the correlation between DECs and their host genes in maternal plasma seems to be slightly confused (the data not shown) (28). Furthermore, given that the complex biofunctions of circRNAs usually are associated with their target miRNAs, co-expressed mRNAs, and binding to RBPs, we annotated the potential functions of key circRNAs in all tissues from these three aspects (29). In maternal plasma, the co-expressed mRNAs of DECs were enriched in the regulation of signaling receptor activity, blood coagulation, and interferon alpha/beta signaling, while 365 miRNAs most likely to be bound by DECs were screened and functional enrichment analysis showed that they were mainly involved in SMAD protein complex assembly, regulation of Wnt signaling pathway, and chemokine receptor activity (**Figures 2D, E**). Moreover, there were 137 RBPs that may interact with DECs (**Figure 2F**). The top 10 RBPs were selected based on node connectivity and they were mainly involved in the metabolism of RNA, particularly IGF2BP2 (**Figure 2G**).

### Key circRNAs and their potential functions in maternal monocytes of PTB

**Figure 3:**
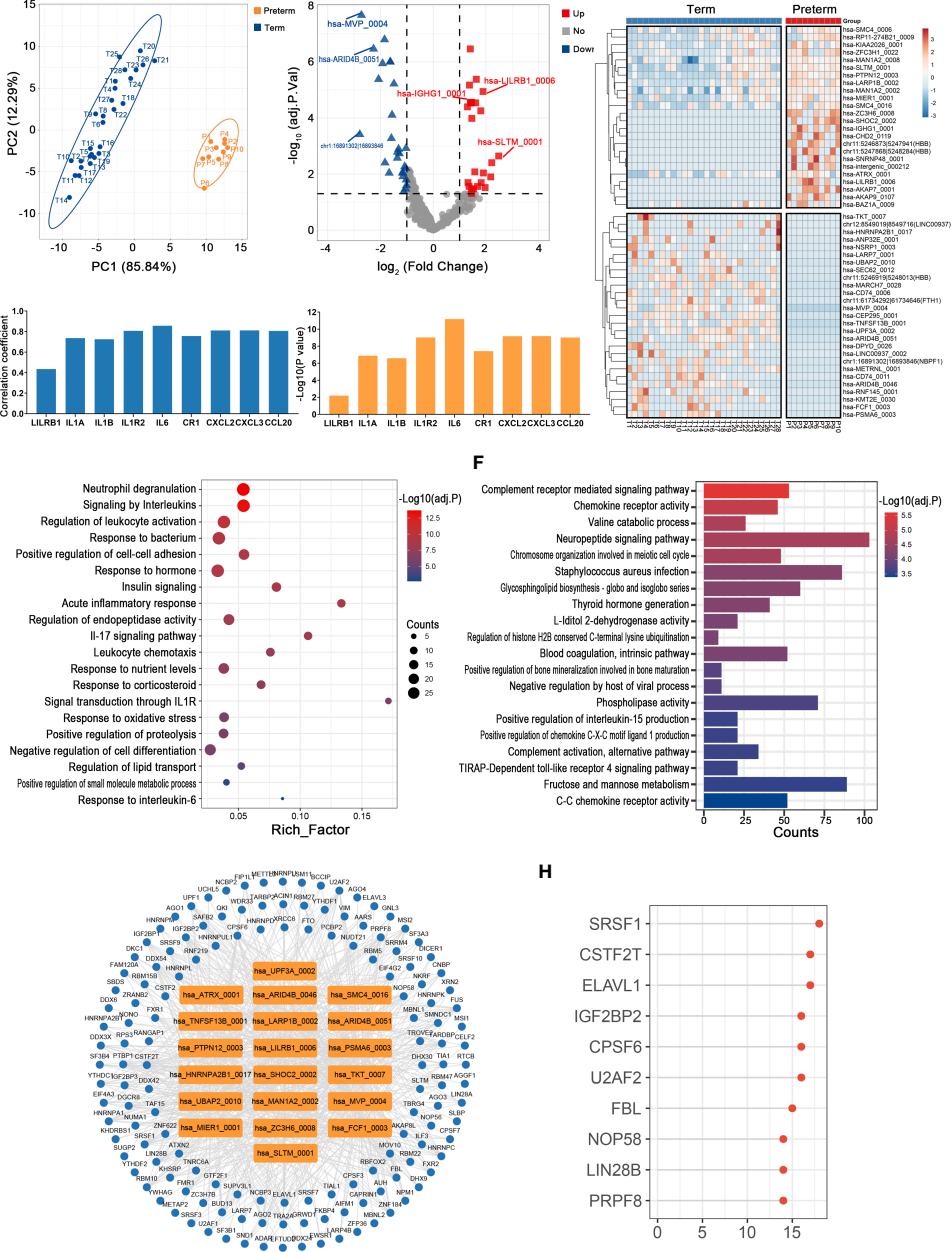
The circRNA changes and functional analyses in maternal monocytes of PTB. **(A)** PLS-DA of circRNA expression in preterm (yellow points) and term (blue points) maternal monocytes. **(B)** The volcano plot of circRNA changes and adj. p-values in maternal monocytes of PTB. **(C)** The hierarchical clustering of DECs expression in term and preterm maternal monocytes. (DECs: |log2 (fold change)|> 1 and adj. p-value < 0.05). **(D)** Correlation coefficients and P values for hsa-LILRB1_0006 and its host gene and several immune inflammatory factors. **(E, F)** Functional enrichment analysis for co-expressed mRNAs **(E)** and target miRNAs **(F)** of DECs in preterm maternal monocytes. **(G, H)** The DECs-RBPs network and top 10 RBPs in maternal monocytes.

Monocytes, as the major source of inflammatory mediators, are essential for the regulation of maternal-fetal immune-inflammatory homeostasis, so they play crucial roles in the pathology of PTB (30). There was clear discrimination in circRNA expression of maternal monocytes between preterm and term pregnancies (**Figure 3A**). A total of 24 up-regulated and 27 down-regulated circRNAs were identified, with hsa-SLTM_0001 (log2FC = 2.48, adj. p < 0.001) and chr1:16891302|16893846 (log2FC = -2.78, adj. p < 0.001) having the most significant fold changes (**Figures 3B, C**) (**Supplementary Table 2**). Among them, hsa-LILRB1_0006 was significantly correlated with its host gene and several immune inflammatory factors, including IL1B and IL6 (**Figure 3D**).

The possible functions of key circRNAs in the maternal monocytes of PTB were inferred using methods consistent with those in maternal plasma. Firstly, 197 co-expressed mRNAs of DECs were selected, which were mainly involved in signaling by interleukins, acute inflammatory response, and leukocyte chemotaxis (**Figure 3E**). Secondly, the DECs might affect the complement, chemokine, and infection through sponging their 745 downstream miRNAs (**Figure 3F**). Thirdly, the circRNA-RBP interaction network containing 20 circRNAs and 144 RBPs was constructed, with the top 10 RBPs represented by SRSF1 (**Figures 3G, H**).

### Key circRNAs and their potential functions in the myometrium of PTB

**Figure 4:**
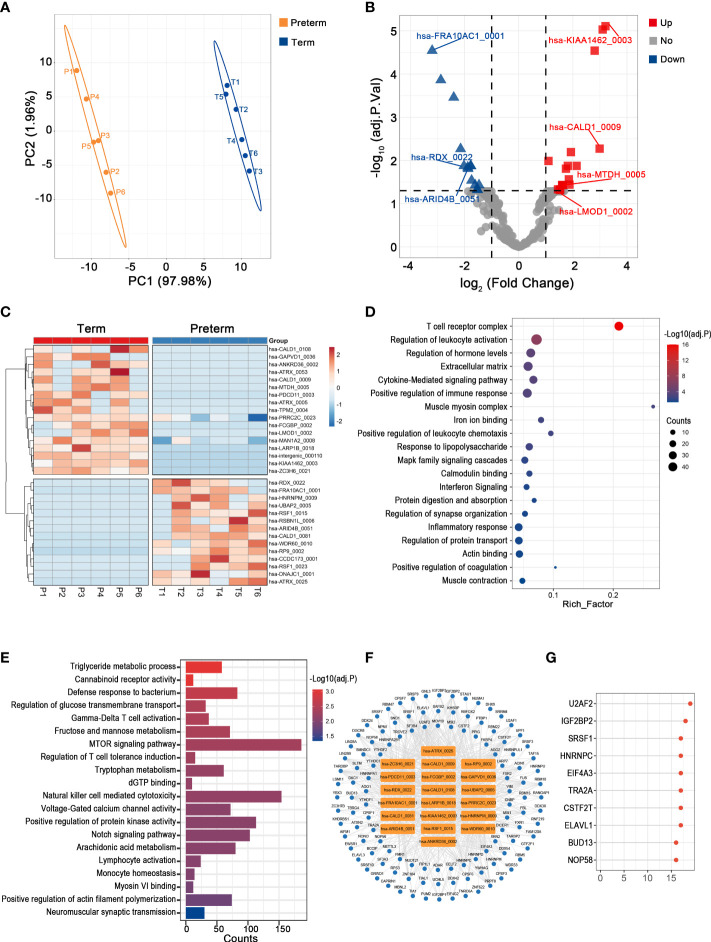
The circRNA changes and functional analyses in the myometrium of PTB. **(A)** PLS-DA of circRNAs expression in preterm (yellow points) and term (blue points) myometrium. **(B)** The volcano plot of circRNA changes and adj. p-values in the myometrium of PTB. **(C)** The hierarchical clustering of DECs expression in term and preterm myometrium. (DECs: |log2 (fold change)|> 1 and adj. p-value < 0.05). **(D, E)** Functional enrichment analysis for co-expressed mRNAs **(D)** and target miRNAs **(E)** of DECs in the preterm myometrium. **(F, G)** The DECs-RBPs network and top 10 RBPs in the myometrium.

Uterine activation and myometrium contraction are markers of labor initiation and the essential basis for PTB. In the myometrium, we observed a significant difference in circRNAs expression between preterm and term groups (**Figure 4A**). Here, the levels of 31 circRNAs were dramatically altered in PTB patients, including 17 up-regulated and 14 down-regulated ones (**Figures 4B, C**) (**Supplementary Table 3**).

The mRNAs that strongly correlated with these DECs were mainly involved in leukocyte activation, cytokine signaling, and muscle contraction (**Figure 4D**). Meanwhile, they competitively bind to 230 downstream miRNAs that may mediate energy metabolism, immune activities, and muscle cell activities (**Figure 4E**). Furthermore, they may interact with 118 RBPs, with the top two being U2AF2 and IGF2BP2 (**Figures 4F, G**).

### Key circRNAs and their potential functions in chorion of PTB

**Figure 5:**
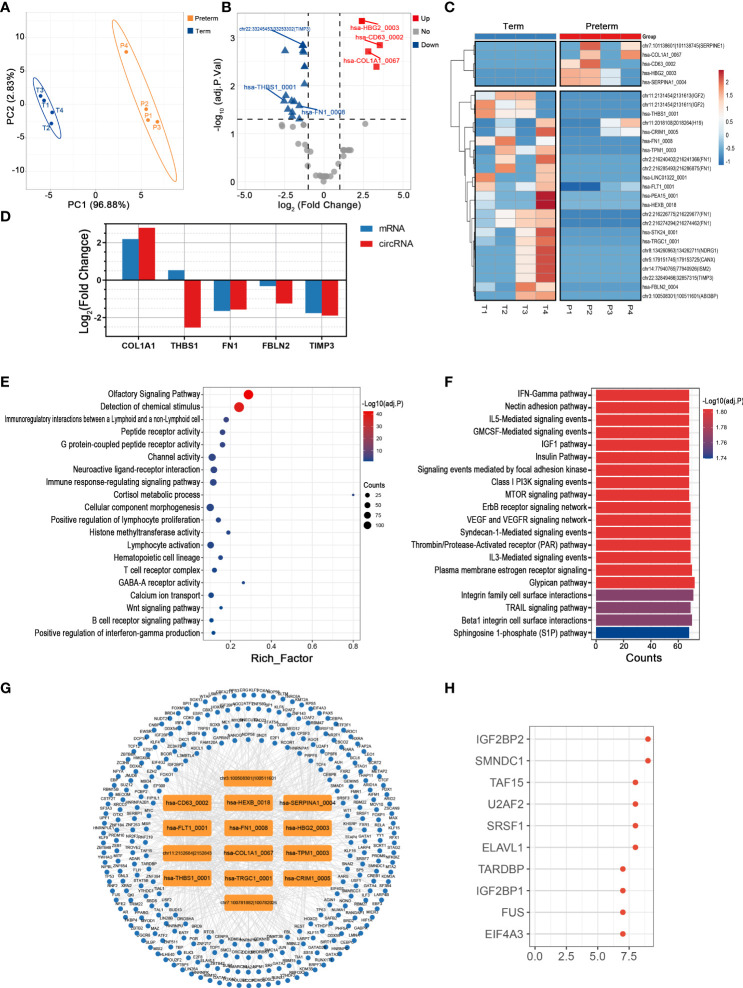
The circRNA changes and functional analyses in the chorion of PTB. **(A)** PLS-DA of circRNAs expression in preterm (yellow points) and term (blue points) chorion. **(B)** The volcano plot of circRNA changes and adj. p-values in the chorion of PTB. **(C)** The hierarchical clustering of DECs expression in term and preterm myometrium. (DECs: |log2 (fold change)|> 1 and adj. p-value < 0.05). **(D)** The fold changes of several specific DECs and their corresponding mRNAs in the preterm compared to the term group. **(E, F)** Functional enrichment analysis of co-expressed mRNAs **(E)** and target miRNAs **(F)** of DECs in the preterm chorion. **(G, H)** The DECs-RBPs network and top 10 RBPs in the chorion.

As the outermost layer of the fetal membrane, the chorion is rich in collagen, and its weakening and rupture indicate the initiation of parturition (31). Since it is directly attached to the inner uterine wall as part of the maternal-fetal interface, it is thought to play an important role in maternal-fetal immune tolerance necessary for pregnancy maintenance through its wide and diverse biological activity. Here, changes in circRNAs between preterm and term groups appeared to be as remarkable as that in the above maternal tissues, consistent with the complex bioreactions and molecular alterations in chorion (**Figure 5A**). Specifically, there were 5 up-regulated and 23 down-regulated circRNAs, with hsa-CD63_0002 (log2FC = 3.53, adj. p = 0.001) and hsa-THBS1_0001 (log2FC = -4.48, adj. p = 0.021) being the most significantly altered circRNAs (**Figures 5B, C**) (**Supplementary Table 4**). We noticed that about one-third of DECs were derived from the extracellular matrix (ECM)-associated genes, such as COL1A1, FN1, and TIMP3. The expression levels of both these DECs and their host genes were disturbed in PTB (**Figure 5D**).

From an overall perspective, the co-expressed mRNAs of DECs were mainly involved in G protein-coupled peptide receptor activity, immune response-regulating signaling pathway, and calcium ion transport (**Figure 5E**). And the 123 miRNAs sponged by these DECs largely associated with interleukin signaling, hormone activities, and cell adhesion (**Figure 5F**). Furthermore, among the top 15 DECs, 14 except chr11:2018108|2018264 had extensive interactions with a total of 285 RBPs (**Figure 5G**). In this network, DECs affected by IGF2BP2 were the most (**Figure 5H**).

### Key circRNAs and their potential functions in the placenta of PTB

**Figure 6:**
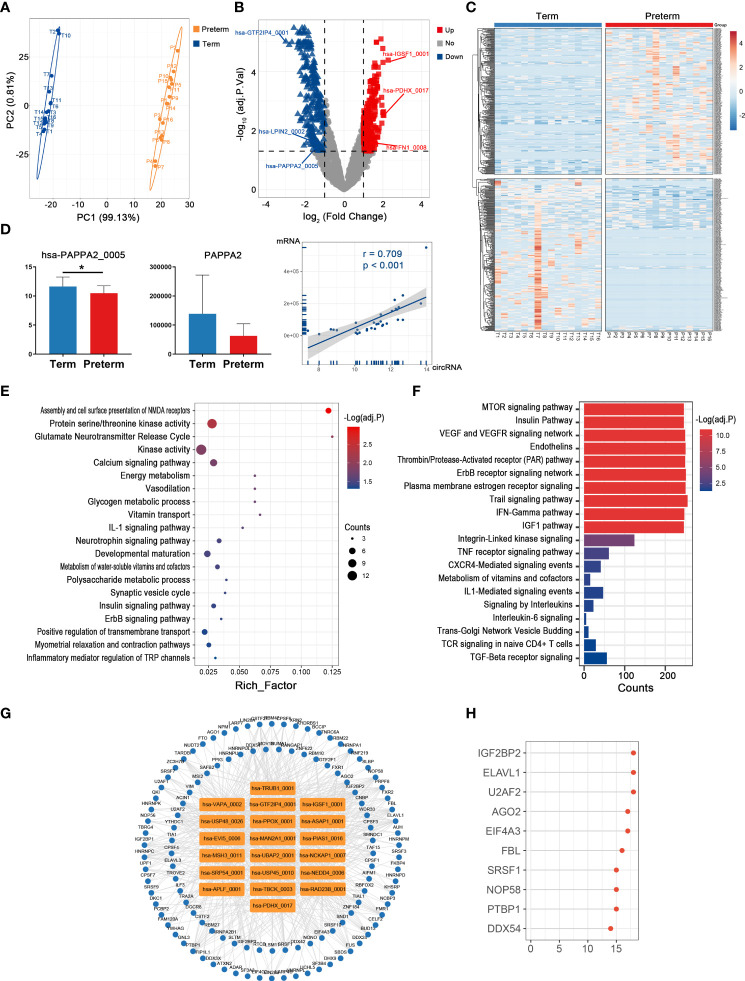
The circRNA changes and functional analyses in the placenta of PTB. **(A)** PLS-DA of circRNA expression in preterm (yellow points) and term (blue points) placenta. **(B)** The volcano plot of circRNA changes and adj. p-values in the placenta of PTB. **(C)** The hierarchical clustering of DECs expression in term and preterm placenta. (DECs: |log2 (fold change)|> 1 and adj. p-value < 0.05). **(D)** The expression levels of hsa-PAPPA2_005 and its host gene PAPPA2 between term and preterm placentas and their correlation. (* indicate p < 0.05) **(E, F)** Functional enrichment analysis for co-expressed mRNAs **(E)** and target miRNAs **(F)** of DECs in the preterm placenta. **(G, H)** The DECs-RBPs network and top 10 RBPs in the placenta.

Given that placenta is the sole organ mediating the material exchange and signaling transmission between the mother and fetus, its intrinsic biological changes are inextricably linked to the process and outcome of pregnancy (32). Here, the pattern of circRNAs expression in the preterm group was significantly different from that in controls (**Figure 6A**). In total, 572 circRNAs were identified as the PTB-related molecules, including 283 up-regulated ones represented by hsa-IGSF1_0001 (log2FC = 2.27, adj. p < 0.001) and 289 down-regulated ones represented by hsa-GTF2IP4_0001 (log2FC = -2.99, adj. p < 0.001) (**Figures 6B, C**) (**Supplementary Table 5**). Notably, the levels of hsa-PAPPA2_005 and its host gene PAPPA2 [an important factor for placental development and function (33)] showed a significant linear positive correlation (p < 0.001), and both were relatively low in PTB (**Figure 6D**).

Based on their 323 co-expressed genes, these 20 top DECs may have an impact on various processes such as energy metabolism, inflammatory process, and development (**Figure 6E**). In addition, by targeting 275 miRNAs, these DECs may affect plasma membrane estrogen receptor signaling, signaling by interleukins, and VEGF and VEGFR signaling network (**Figure 6F**). In the circRNA-RBP network, these DECs interacted with 115 RBPs, and the top one was IGF2BP2 (**Figures 6G, H**).

### Key circRNAs and their potential functions in the cord blood of PTB

**Figure 7:**
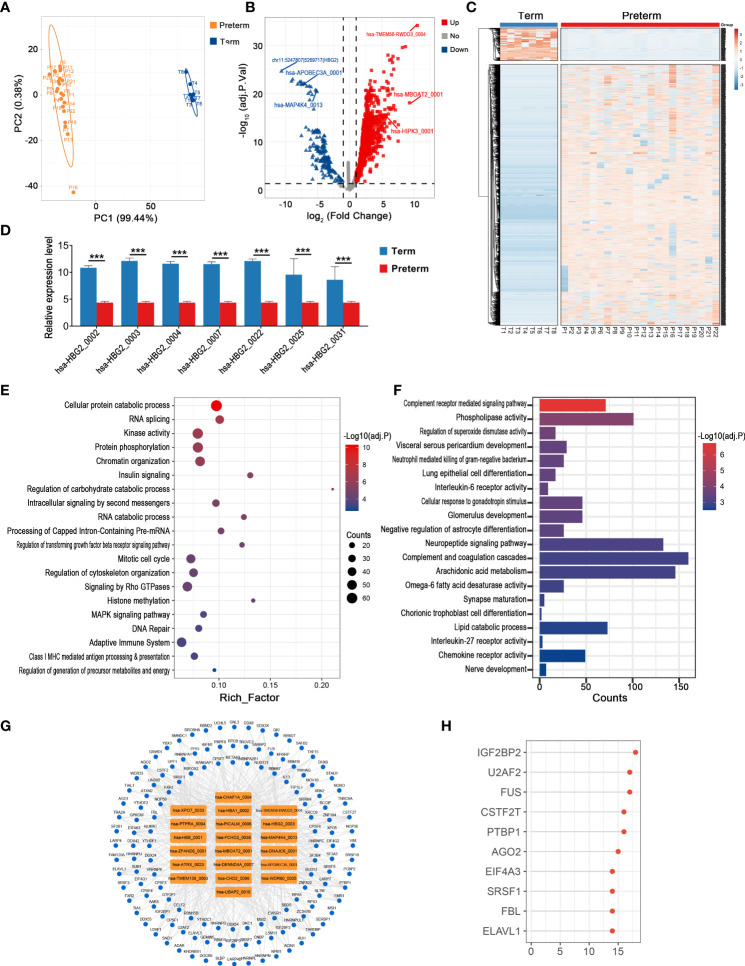
The circRNA changes and functional analyses in the cord blood of PTB. **(A)** PLS-DA of circRNA expression in preterm (yellow points) and term (blue points) cord blood. **(B)** The volcano plot of circRNA changes and adj. p-values in the cord blood of PTB. **(C)** The hierarchical clustering of DECs expression in term and preterm cord blood. (DECs: |log2 (fold change)|> 1 and adj. p-value < 0.05). **(D)** The expression levels of DECs originated from HBG2 in preterm and term cord blood. **(E, F)** Functional enrichment analysis for co-expressed mRNAs **(E)** and target miRNAs **(F)** of DECs in preterm cord blood. **(G, H)** The DECs-RBPs network and the top 10 RBPs in the cord blood. (*** indicate p < 0.001).

The cord blood provides access to information on systemic physiological and pathological changes in the fetus. In terms of the overall circRNA expression in cord blood, individual variability was greater within the preterm group compared to the term group, while there was still a clear distinction between these two groups (**Figure 7A**). In detail, most DECs were up-regulated in the preterm group, 1527 in total, with hsa-TMEM56-RWDD3_0004 being the one with the largest fold change (log2FC = 10.51, adj. p < 0.001). Meanwhile, there were 192 down-regulated circRNAs and the top one was a novel circRNA derived from intergenic_region, chr11:5247807|5269717 (log2FC = -10.62, adj. p < 0.001) (**Figures 7B, C**) (**Supplementary Table 6**). Interestingly, we found several circRNAs from HBG2 were significantly downregulated in PTB (**Figure 7D**).

We selected the top 10 up-regulated and 10 down-regulated circRNAs to further explore the possible biological impacts of circRNAs disorders in cord blood. Firstly, 1126 co-expressed mRNAs of these DECs were enriched in DNA replication, RNA splicing, adaptive immune system, and so on (**Figure 7E**). Secondly, these key circRNAs affected nerve development, complement, and interleukin signaling by binding to 429 miRNAs (**Figure 7F**). Thirdly, these DECs provided binding sites for 136 RBPs, and the most significant one remained IGF2BP2, which was the same as the result in maternal plasma, myometrium, chorion, and placenta (**Figures 7G, H**).

### The overall characteristics of DECs and their roles in the PTB

**Figure 8:**
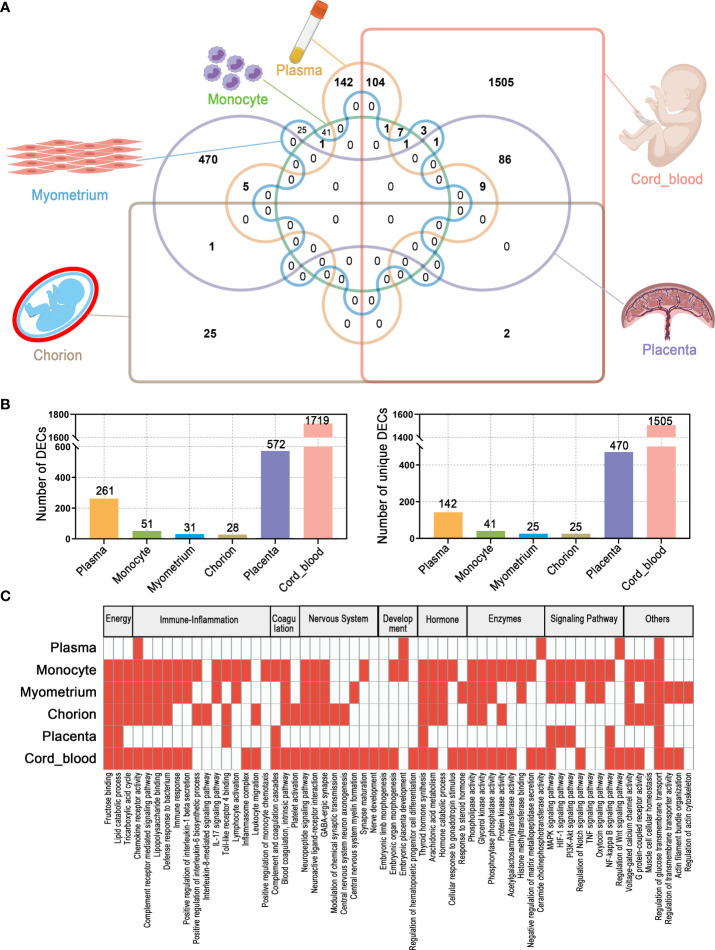
The overall characteristics of DECs and their possible functions in multiple maternal-fetal tissues of PTB. **(A)** Venn diagram of DECs in different tissues. **(B)** The number of total DECs and unique DECs in different tissues. **(C)** Summary and comparison of potential biological functions of DECs among different tissues.

Altogether, circRNA alterations associated with PTB were observed from the maternal circulation to the maternal-fetal interface to the fetal circulation. The number of DECs varied with tissue type, with the highest number of 1719 in cord blood, while the lowest number in the chorion, only 28. Notably, there were some shared DECs across tissue types, for example, up to 104 circRNAs dysregulated in both maternal and fetal circulation in PTB (**Figures 8A, B**) (**Supplementary Table 7**). Although the potential roles of DECs in different tissues of PTB were extremely complex and diverse, the key DECs in these tissues might were involved in several shared biological processes and reactions, such as energy metabolism, immune-inflammatory responses, coagulation, nervous system activities, development, hormonal synthesis and secretion, enzyme activity, and signaling pathways. In particular, the classical PTB-related immune-inflammatory processes were significantly associated with the top DECs in all tissues (**Supplementary Figure 2**). Of course, the expression and putative functions of DECs in different tissues were also linked to the intrinsic characteristics of the tissues. For example, the myometrium, as a target and executor of various delivery signals, is mainly responsible for contractile functions, and therefore, the number of DECs in it is relatively small and the molecular mechanisms affected by DECs are relatively simple (**Figure 8C**).

## Discussion

PTB is a syndrome that seriously impairs the health of the mother and fetus (4). Understanding the systemic molecular changes in PTB is an important basis for investigating its pathogenesis and predictors. In this study, for the first time, we comprehensively analyzed the circRNA expression patterns in multiple tissues within the maternal-fetal system of PTB, including maternal plasma, maternal monocytes, myometrium, chorion, placenta, and cord blood, and further predicted potential biofunctions of key circRNAs from multiple perspectives. Altogether, our research provides an overview of the circRNA expression landscape and its putative biological roles in PTB.

To a holistic insight, the expression of circRNAs in maternal tissues, maternal-fetal interface, and fetal circulation of PTB were significantly changed in comparison with that of controls, suggesting that systematic changes of circRNAs were involved in the pathogenesis of PTB. It is noteworthy that some DECs were shared by different tissues, suggesting that there might be identical pathological mechanisms dominated by circRNAs in these tissues, and that these circRNAs may mediate inter-tissue or local-to-systemic signaling communications in PTB. For example, hsa-HIPK3_0001 (circHIPK3) was up-regulated both in maternal plasma and cord blood of PTB. Thus, to predict the potential roles of circRNAs in PTB, we identified the target miRNAs, co-expressed mRNAs, and RBPs which were bound to or regulated by DECs, and performed functional annotation. As previously described, circRNAs are traditionally thought to regulate various biological processes by acting as miRNA sponges and gene expression regulators (8, 34, 35). On this basis, we found that the biological activities associated with DECs in different tissues have their own characteristics, which may be explained by the intrinsic properties and functions of the tissues. For instance, the DECs in maternal monocytes were mainly focused on immune inflammation, while those in the myometrium were related to positive regulation of cellular contractility, and those in cord blood mainly participated in the development. Nevertheless, the immune-inflammatory response remains specific and prominent (e.g., interleukin 1/6/8/17 signaling pathway, chemokine, complement, and TLRs binding). As one of the recognized core mechanisms of PTB, we found that DECs in different maternal-fetal tissues were extensively associated with it (36, 37). This suggested that DECs may play important synergistic roles in PTB through these common pathways in different tissues. Recently, the emerging circRNA-RBP interactions have been demonstrated to be involved in physiological and pathological mechanisms of diseases (7, 38, 39). On one hand, RBPs are acknowledged as key post-transcriptional regulators that influence the level and function of circRNAs by participating in their biogenesis, stabilization, and localization (40). For example, the RBP EIF4A3 inhibits the formation of circRNA_100290 by binding to it, and in gastric cancer, dysregulation of this regulatory mechanism would lead to the increased level of this circRNA, which triggers downstream activities such as tumor cell proliferation (41). On the other hand, circRNA influences the regulation of RBP on downstream target genes by acting as a sponge, assembly platform, or transport vector for it (40). For example, in ovarian cancer, circE2F2 competitively binds to HuR protein, thereby attenuating its contribution to E2F2 mRNA stability, leading to disruption of E2F2 levels and its participating activities such as cell proliferation and migration (42). Here, we revealed for the first time the network of DECs-RBPs within various maternal-fetal tissues in PTB and found that IGF2BP2 is the most conspicuous RBP in all these networks. This suggested a non-negligible role of RBP in circRNA-mediated pathogenesis of PTB, which is possible through the above ways (43, 44). As a member of the human IGF2 mRNA binding protein family, IGF2BP2 is involved in a wide range of biological processes such as development, metabolism, tumorigenesis, and immunity through post-transcriptional regulation of a variety of genes (45). Notably, IGF2BP2 not only could promote immune and inflammatory responses by activating macrophages and targeting caspase 4 (46, 47), but also regulates the expression and epigenetic modification of IGF2 (45, 48), both of which have been proven to be associated with PTB (36, 49, 50). More importantly, it has been reported that IGF2BP2 could mediate the production of circRNAs and interact with circRNAs in multiple biological activities (44, 51). For example, circNDUFB2 could facilitate the ubiquitination and degradation of IGF2BP2 to modulate cellular immune responses in non-small cell lung cancer (51). In turn, IGF2BP2 modifies circRNA circARHGAP12 and enhances its ability to bind and stabilize the oncogene FOXM1, thus promoting tumor cell proliferation and migration in cervical cancer (44). Based on the above evidence, we speculated that the interactions between circRNAs and IGF2BP2 might have effects on multiple aspects represented by the immune-inflammatory response, and coordinate the molecular activities of the various tissues in PTB.

In samples from maternal circulation (plasma and monocytes), we found that the PTB-related DECs might contribute to the pathological process of PTB by involving the immune-inflammatory response, which is in agreement with our previous finding in peripheral whole blood (5). In maternal plasma, 33 DECs were identical to those previously identified in peripheral whole blood, and 18 of them showed the same change pattern in both previous and current studies. More interestingly, circRNAs from the homeodomain-interacting protein kinase (HIPK) family were significantly increased in maternal peripheral blood, maternal plasma, and cord blood of PTB. In this study, hsa-HIPK3_0001 (circHIPK3) is one of the up-regulated DECs both in maternal plasma and cord blood, which has been reported as an inflammatory regulator in numerous diseases (52–54). For example, increased circHIPK3 stimulated the activation of the TLR4 pathway and NLRP3 inflammasome and the production of pro-inflammatory cytokines (e.g., TNF-α, IL-1β) in gouty arthritis (54) whereas its silencing alleviated inflammatory damage in myocarditis (53) and diabetes mellitus (52). Considering that immune-inflammatory imbalance is the indispensable pathogenic process of PTB (4, 55, 56), we proposed that abnormally elevated circHIPK3 may aggravate the progression of PTB by causing excessive inflammation. Furthermore, we found that PTB-related DECs in maternal plasma may affect the chemokine receptor activity, which has been illustrated to be closely associated with inflammation-induced myometrial contraction activation and membrane rupture in PTB (57–59). In a word, the above findings suggested that altered circRNAs in maternal plasma of PTB might be the mediators of its immune-inflammatory mechanism. Then, this speculation was further corroborated in maternal circulating monocytes. As a key executor of innate immunity, the monocyte is one of the earliest responding cells during the process of immune-inflammatory imbalance. It is rapidly activated and proliferates after receiving pathological signals, thus producing a large number of pro-inflammatory factors (e.g. IL-1β, TNF-α) to activate inflammatory signaling pathways (e.g. NF-κB) in various tissues and organs throughout the body as well as a full range of complement proteins to amplify the inflammatory effects, while infiltrating maternal-fetal interface tissues across the vascular wall under the stimulation of chemokines, causing progressively increasing inflammatory damage and thus leading to PTB (30, 60–65). Coincidentally, we found that PTB-related DECs in monocytes were mainly involved in this complex series of immune-inflammatory responses, including NF-kappa B signaling pathway, complement receptor-mediated signaling pathway, chemokine receptor activity, and interleukin signaling. In fact, the NF-κB signaling which is regulated by circRNAs not only controls the activation and differentiation of monocytes, but also mediates their effects on inflammation induction in target tissues (66). Recently, it has been demonstrated that excess circRNA circPPM1F causes pancreatic islet inflammation by activating monocytes/macrophages, which is achieved by competitively binding RBP HuR (an important regulator of gene expression) to attenuate the inhibition of NF-κB by the downstream gene PPM1F (67). And circRNA circGLIS2 was also found to enhance tumor cell motility and pro-inflammatory chemokine secretion by activating NF-κB in colon cancer (68). Therefore, we speculate that these DECs might act as essential mediators contributing to monocyte activation, migration to the maternal-fetal interface, and pro-inflammatory function in PTB. Moreover, monocytes are a remarkable source of complement proteins, and these two act synergistically to promote each other in inflammation progression to trigger uterine contractions, membrane disruption, and cervical ripening in PTB, while we found that circRNA in monocytes might influence their production of or response to complement. Thus, despite the lack of reports on the relationship between circRNA and complement (especially in monocyte), this might indeed be a new and critical link in the inflammatory mechanism of PTB that deserves further exploration. Overall, there were circRNA disorders and they mainly lead to the disruption of immune-inflammatory balance in the maternal circulation of PTB.

The maternal-fetal interface, including the myometrium, chorion, and placenta, forms the connection between mother and fetus throughout pregnancy (69). The dysregulated circRNAs between the preterm and term groups varied among these tissues. Compared to the placenta, DECs in the myometrium were fewer and their potential functions were mainly limited to positive regulation of cellular contractility, which is consistent with strong uterine contractions during parturition (58, 70, 71). While the circRNA changes and the functions involved in the chorion were much more diverse and active than that in the myometrium, which is probably due to its role as the forefront of fetal tissue exposure to the mother, where intrinsic molecular responses are complex and active, whereas the myometrium is the downstream effector tissue with fewer molecular and signaling alternations. In chorion, we found that several DECs originated from the genes associated with ECM. As described above, circRNAs could regulate the expression of their host genes, the functions of these two have a degree of similarity (28), which means that we can annotate the impacts of circRNAs on PTB *via* analyzing their host genes. It is well known that damage and rupture of the fetal membrane will cause PTB, and the maintenance of its normal structure is mainly dependent on the collagen-rich ECM. And the host genes of PTB-related circRNAs we identified have essential effects on collagen and ECM. Firstly, COL1A1 and FN1 are the genes that encode the component of the fetal membrane ECM, so their dysregulation directly reflects the abnormalities of the basic biological structure of the fetal membrane (72). Secondly, inflammation and MMPs cause the degradation of ECM and collagen, resulting in membrane weakening and rupture (73–77). TIMP3 is an inhibitor of MMPs and the balance of TIMPs/MMPs is the necessary condition for maintaining the normal ECM remodeling process, while their imbalance would lead to the destruction of the internal structure of the ECM and trigger PTB (78–80). In addition, THBS1 could induce immune inflammation through downstream TGF-β and NF-κB pathways, which further facilitate the structural disruption of the maternal-fetal interface (81, 82). Thus, the DECs derived from the above genes might mediate the essential processes of ECM remodeling and collagen degradation in PTB. Since it is generally accepted that pro-inflammatory responses and immune tolerance breaking are the important causes of fetal membrane damage and rupture, uterine contractions, and cervical changes, thus resulting in the occurrence of PTB, we speculated that the DECs in the chorion might regulate inflammatory processes in the development of PTB (83–86). The evidence was provided by the target miRNAs and co-expressed mRNAs. By acting them, DECs in chorion were involved in multiple inflammatory signaling pathways, including interleukin signaling, chemokine activity, complement, and TLRs, consistent with the above results in maternal circulation (plasma and monocyte). This suggested that dysregulated circRNAs at the maternal system and maternal-fetal interface may collectively contribute to the inflammatory response in PTB through these common pathways. The placenta, as another component of the maternal-fetal interface, is the major bridge of the material exchange and signaling transmission between mother and fetus. Thus, its metabolism is highly active and complex, which is necessary for maintaining the progression of pregnancy, and its disruption often leads to maternal-fetal complications, such as PE, FGR, and PTB (32, 87). The PTB-related DECs we found in the placenta were markedly involved in energy metabolic activities, especially lipid catabolic process, in addition to regulating inflammatory signaling pathways shared with other tissues. Fatty acid metabolism is an important part of energy metabolism, and fatty acid metabolites have long been recognized as the important mediators of the pathological inflammatory responses in PTB. For instance, short-chain fatty acids (SCFAs) butyrate and propionate could suppress the pro-inflammatory cytokines and prostaglandin signaling through the MAPK pathway, thereby inhibiting uterine contractions in PTB (88). Besides, the excess pro-inflammatory free fatty acids (FFAs), as well as reduced omega-3 fatty acids with anti-inflammatory effects, have been shown to contribute to uncontrolled inflammation and membrane rupture in PTB (89). Currently, circRNAs are starting to be reported that could regulate the fatty acid metabolism in diseases such as non-alcoholic fatty liver disease (NAFLD) and cancer, mainly by affecting key enzymes and related molecular signaling pathways in their metabolic processes (90, 91). However, studies of circRNA and fatty acids in the specific and complex biological context of pregnancy are lacking, and our study might provide a new entry point for this. Integratively, circRNAs were significantly altered at the maternal-fetal interface of PTB, and in addition to their specific roles in different tissues (e.g., cellular contraction in the myometrium, ECM degradation in the chorion, fatty acid metabolism in the placenta), they are also collectively involved in the immune-inflammatory response. This is consistent with the findings in the maternal tissues, further emphasizes the essential roles of inflammation in the pathogenesis of PTB and suggests the potential association and synergy between maternal systemic and local inflammation at the maternal-fetal interface.

In addition to maternal circRNAs changes, the expression of circRNAs in preterm neonates also differed significantly compared to term controls. Firstly, in cord blood, six down-regulated DECs derived from HBG2, which is closely associated with fetal hemodynamics, showed similar patterns of change (92). The HBG2 encodes the hemoglobin subunit gamma-2 protein, the predominant type of hemoglobin in the fetus, which is responsible for oxygen transport within the circulation, so the disorder of this gene would result in fetal hematological abnormalities (e.g., anemia, cyanosis, hypoxia) (92). Therefore, we speculated that the significant reduction in these circRNAs derived from HBG2 may partially explain the higher incidences of cyanosis and hypoxia in preterm babies (93). Secondly, we observed that DECs in cord blood partially overlap with that in maternal circulation and may affect immune-inflammatory processes shared with maternal DECs, such as complement and NF-κB pathways. Previous studies have reported that excessive activation of the fetal immune system and overproduction of pro-inflammatory cytokines (e.g., IL-6, IFN-γ, TNF-α) in fetal circulation could stimulate inflammatory outbreaks at the maternal-fetal interface and maternal system, leading to fetal membrane rupture and uterine contractions, then triggering the onset of PTB (55, 94, 95). Thus, circRNAs might not only mediate inflammation response in mothers, but also promote that in the fetus in PTB, and there may be potential interaction and communication between them. Nevertheless, further studies are needed to investigate whether the fetal inflammatory storm in PTB and the fetal-maternal inflammatory communication are mediated by fetal or/and maternal circRNAs. Thirdly, DECs in cord blood may additionally be engaged in neurodevelopment, which is directly associated with marked cerebral palsy as well as lethal cerebral hemorrhage in preterm babies. It is well-known that circRNAs are highly abundant and spatiotemporally changed in the nervous system, and were the crucial controllers and effectors in the neurodevelopment and the pathogenesis of neurological disorders (96, 97). Recently, notable disorders of circRNAs associated with neurogenesis, neuronal development, and neural regeneration and repair have been identified in the circulation of premature infants with periventricular white matter damage (98), which supports our findings to some extent. Considering this evidence and the high prevalence of neurological damage in premature babies (99), we suggested that DECs we found in cord blood may be implicated in the underlying mechanisms of neurological injury, which would bring new opportunities to develop therapeutic targets for improving neurological outcomes of preterm babies.

Overall, circRNAs expression is systemically altered in PTB, from the maternal circulation to the maternal-fetal interface to the fetal circulation. DECs in different tissues are overwhelmingly different, but there are still a small number of DECs shared by various tissues, suggesting that circRNA disorders in PTB may constitute multiple molecular networks that are relatively independent yet communicate with each other. From the perspective of circRNA function, we were pleasantly surprised to find that DECs in each maternal-fetal tissue might be all predominantly involved in the recognized key pathological manifestations of that tissue in PTB, such as structural disruption of the chorion, metabolic disorders of the placenta, and dysregulation of oxygen supply of the fetal circulation. This suggests that circRNA may be a novel essential regulatory link in the pathological mechanism of PTB after proteins and linear RNAs, which may provide an important twist and opportunity for the PTB puzzle-solving process. In addition to these tissue-specific roles of circRNAs, we found they may play the same role in these tissues to some extent, mainly involving immune-inflammatory response. Our findings provide a comprehensive understanding of circRNAs expression in PTB and predict their potential roles in PTB pathogenesis, as well as implying the possible mutual communication and co-interaction of circRNA changes and immune inflammation between maternal and fetal systems in the systemic disease, PTB.

Some limitations are however present. Firstly, we identified DECs between preterm and term groups through bioinformatic analysis of RNA-seq datasets from various maternal-fetal tissues, but considering the heterogeneity of different data, there might be a somewhat discrepancy between our results and the actual circRNAs expression levels. Secondly, our study provides the landscape of circRNA and a general understanding of their potential regulatory roles in PTB. Nevertheless, the specific expression and exact roles of circRNAs in local tissues of PTB deserve further validation and in-depth exploration. Therefore, based on our results, targeted studies and complex basic experiments need to be conducted in the future to uncover the specific mechanisms of circRNAs in the pathogenesis of PTB.

## Conclusion

In conclusion, this study explored the systemic changes of circRNAs in multiple maternal-fetal tissues, thus establishing the landscape of circRNAs expression in PTB. Furthermore, we showed that these circRNAs may not only have tissue-specific biological functions and thus are responsible for the hallmark pathological alterations in different maternal-fetal tissues in PTB, but also mediate the communication and collaboration of various tissues within the maternal-fetal system mainly by participating in immune-inflammatory signaling pathways (e.g., complement, interleukin1/6/8/17, chemokine, and TLRs), thus promoting PTB. For the first time, our work formed the overall understanding of circRNAs in PTB. These findings would open up an entirely new area of research in PTB pathogenesis and provide the initial blueprints and necessary basis for further studies.

## Data availability statement

The datasets presented in this study can be found in online repositories. The names of the repository/repositories and accession number(s) can be found in the article/**Supplementary Material**.

## Author contributions

NY and HQ designed the research. YR and RC performed bioinformatic analysis. YR wrote the manuscript. DH, YQ, and YM helped to perform the statistical analysis. ZL, JH, and YZ contributed to the data collection.

## Funding

This work was supported by grants from the Chongqing Municipal Health Commission [No. 2020MSXM029], Chongqing Municipal Education Commission [No. CYB20141], and Science and Technology Department of Sichuan Province [No. 2020YFQ0006].

## Acknowledgments

The authors thank E Gong (Women and Children’s Hospital of Chongqing Medical University), for administrative support. The authors also would like to appreciate the support from “111 program” of Ministry of Education P.R.C and State Administration of Foreign Experts Affairs P.R.C.

## Conflict of interest

The authors declare that the research was conducted in the absence of any commercial or financial relationships that could be construed as a potential conflict of interest.

## Publisher’s note

All claims expressed in this article are solely those of the authors and do not necessarily represent those of their affiliated organizations, or those of the publisher, the editors and the reviewers. Any product that may be evaluated in this article, or claim that may be made by its manufacturer, is not guaranteed or endorsed by the publisher.
