# Supplementary material for: The landscape of circular RNA in preterm birth
Source: Front Immunol. 2022 Aug 22;13:879487. doi: 10.3389/fimmu.2022.879487 (PMC9441874; doi:10.3389/fimmu.2022.879487)
Supplement: Supplementary file 1 [file DataSheet_1.docx]

***Supplementary Figures***


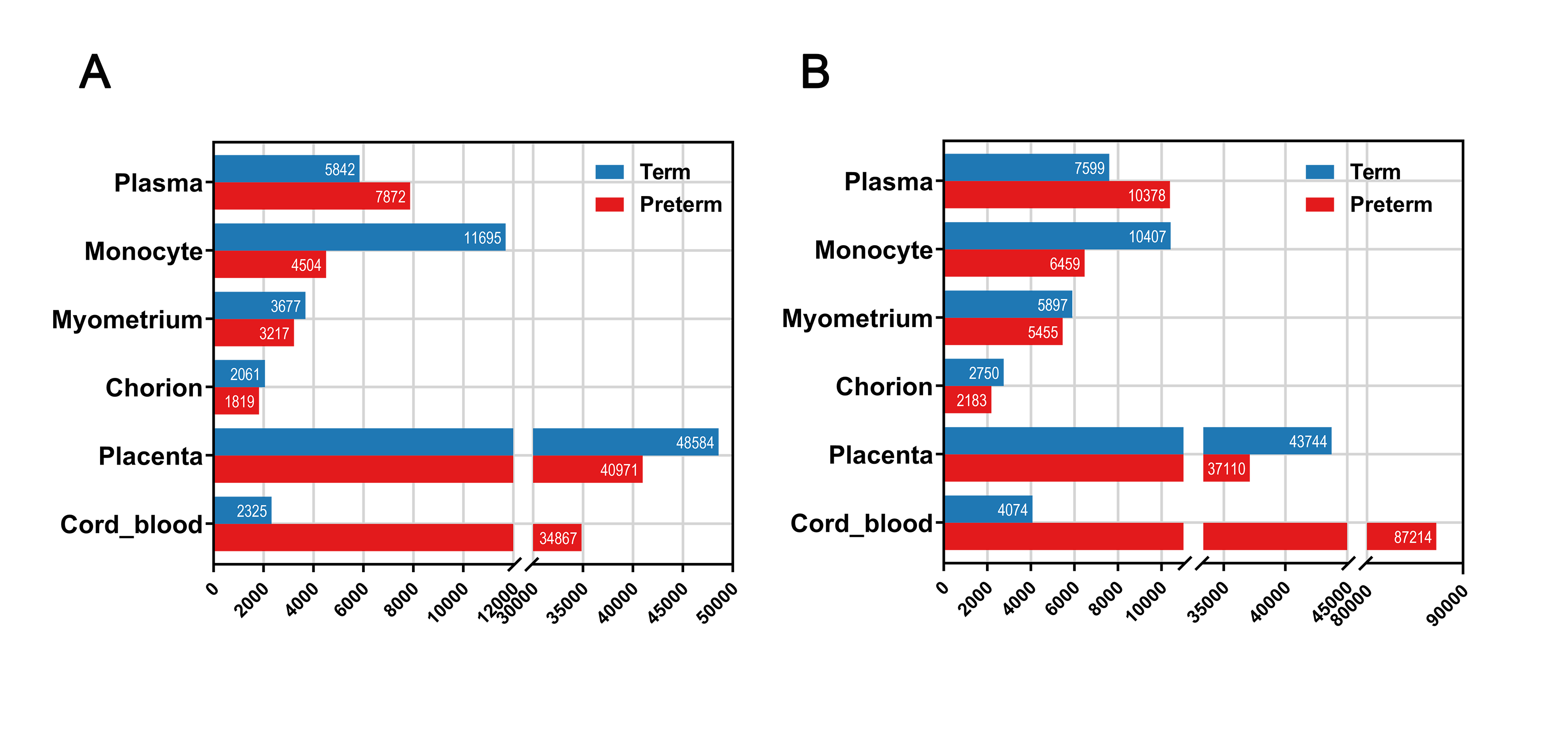


**Supplementary Figure 1.** **The number of circRNAs identified in different groups and tissue types. (A)** The number of circRNAs identified by CIRIquant. **(B)** The number of circRNAs identified by CIRCexplorer2.


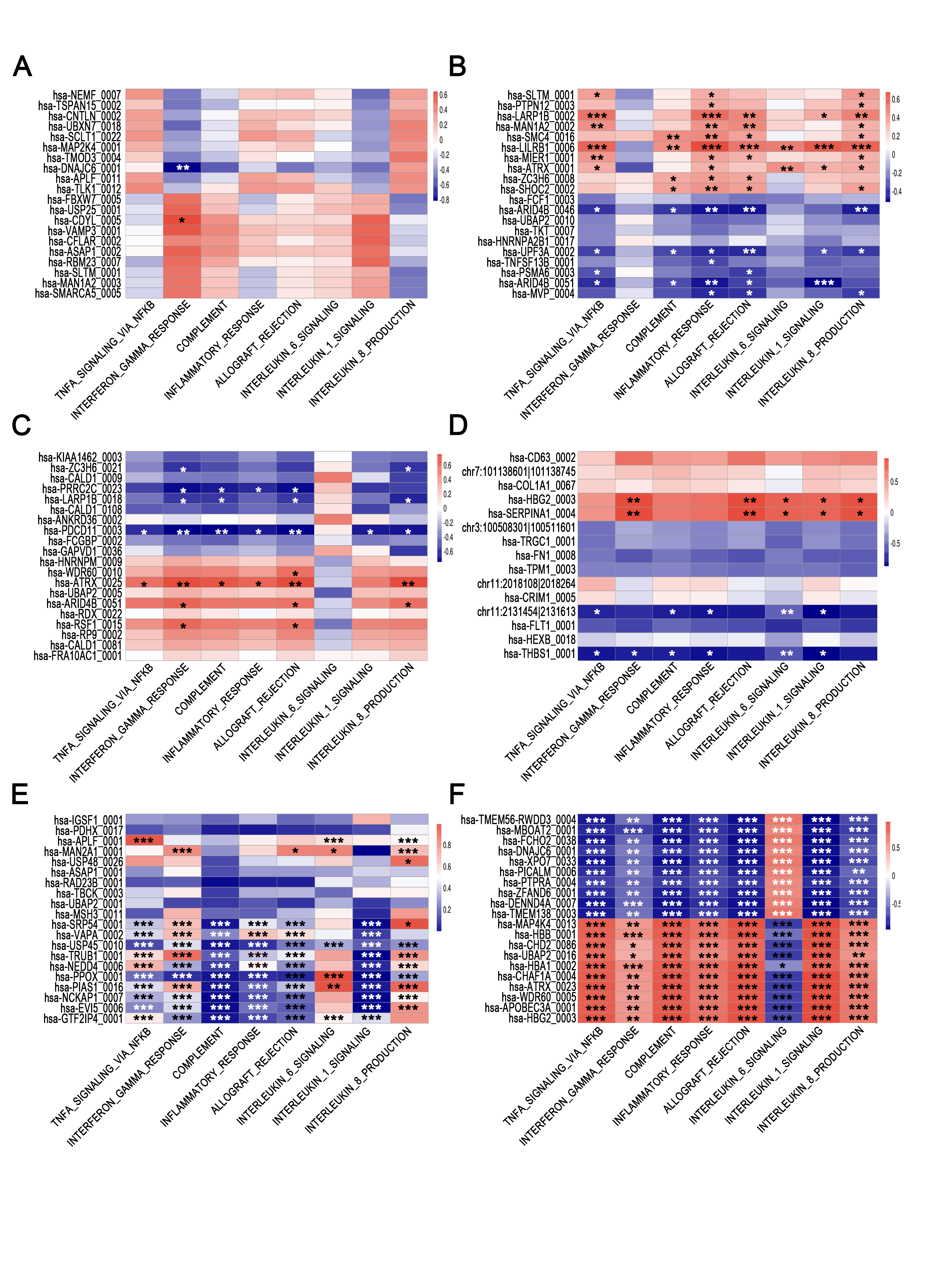


**Supplementary Figure 2.** **Relationships between DECs and classical immune inflammatory processes in different tissues. (A)** Maternal plasma. **(B)** Maternal monocytes. **(C)** Myometrium. (D) Chorion. **(E)** Placenta**. (F)** Cord blood. (The color of heatmap indicate correlation coefficient between GSVA score and DECs expression level; * indicate p < 0.05; ** indicate p < 0.01; *** indicate p < 0.001)
